# Emphasizing the Crosstalk Between Inflammatory and Neural Signaling in Post-traumatic Stress Disorder (PTSD)

**DOI:** 10.1007/s11481-023-10064-z

**Published:** 2023-04-25

**Authors:** Anusha Govindula, Niraja Ranadive, Madhavan Nampoothiri, C Mallikarjuna Rao, Devinder Arora, Jayesh Mudgal

**Affiliations:** 1 Department of Pharmacology, Manipal College of Pharmaceutical Sciences, Manipal Academy of Higher Education, Manipal, Karnataka 576104 India; 2 School of Pharmacy and Medical Sciences, Griffith University, Gold Coast campus, Gold Coast, Queensland, 4222 Australia

**Keywords:** Post-traumatic stress disorder, Neuroinflammation, Serotonin, Glutamate, Cyclooxygenase, Prostaglandins

## Abstract

Post-traumatic stress disorder (PTSD) is a chronic incapacitating condition with recurrent experience of trauma-related memories, negative mood, altered cognition, and hypervigilance. Agglomeration of preclinical and clinical evidence in recent years specified that alterations in neural networks favor certain characteristics of PTSD. Besides the disruption of hypothalamus-pituitary-axis (HPA) axis, intensified immune status with elevated pro-inflammatory cytokines and arachidonic metabolites of COX-2 such as PGE2 creates a putative scenario in worsening the neurobehavioral facet of PTSD. This review aims to link the Diagnostic and Statistical Manual of mental disorders (DSM-V) symptomology to major neural mechanisms that are supposed to underpin the transition from acute stress reactions to the development of PTSD. Also, to demonstrate how these intertwined processes can be applied to probable early intervention strategies followed by a description of the evidence supporting the proposed mechanisms. Hence in this review, several neural network mechanisms were postulated concerning the HPA axis, COX-2, PGE2, NLRP3, and sirtuins to unravel possible complex neuroinflammatory mechanisms that are obscured in PTSD condition.

## Introduction

Post-traumatic stress disorder (PTSD) is a persistent enervative condition that progresses aftermath of a traumatic incident usually after the context of war, sexual violence, and natural disaster (American Psychological Association 2017). According to the 5th version of the Diagnostic and Statistical Manual of Mental Disorders (DSM-V), PTSD comprises four symptom clusters viz., presence of intrusive memories related to a stressful incident, persistent avoidance of stimuli, negative alteration in mood or cognition, and hyperarousal. These symptoms must have been present for one month after experiencing a traumatic event causing significant distress or social impairment. However, the symptoms may also be delayed even after years and the individual might experience a severe crisis later in life (Weathers et al. 2014; American Psychiatric Association 2017).

It is well documented that veterans and members of the armed forces are at greater risk of PTSD (Moore et al. 2021). The primary progenitor of PTSD among war veterans has certainly combated trauma (O’Toole et al. 2020). Around 8% of the general population suffers from posttraumatic stress disorder (PTSD). Veterans and active military personnel both experience PTSD at twice the rate as the general population (Judkins et al. 2020). According to WHO World Mental Health (WMH) surveys in 24 countries, rape (13.1%), sexual assault (15.1%), being stalked (9.8%), and sudden loss of a loved one (11.6%) had the largest proportions of this burden. The first three of these four traumas are very unusual with a high PTSD risk, whereas the fourth is a highly frequent event with a low PTSD risk (Kessler et al. 2017). Furthermore, the prevalence of PTSD in Indian settings varies greatly, from little to almost 70%. The disparity in PTSD prevalence rates has mostly been linked to methodological variations across the research, including variations in the severity of the catastrophe used for the study, technique of sampling, case identification, etc. (Pillai et al. 2016). Notably, a cross-sectional comparative study in Nepal identified strong association between pro-inflammatory cytokines and trauma, confirming the hypothesis of immune system activation in trauma (Koirala et al. 2023). Similarly, Case-control studies in PTSD patients showed high levels of chemokines and proinflammatory cytokines, indicating that these increased inflammatory markers could act as biomarkers of PTSD risk, resilience, and stress responses (Zhang et al. 2020; Otsuka et al. 2021). These upregulated proinflammatory markers imply that those suffering from PTSD may have an activated immune system, which might contribute to neuroinflammation (de Oliveira et al. 2018).

### Neurobiological Mechanisms of Stress-Induced Inflammation in PTSD

Usually, the reaction to physiological and/or psychological stressors involves a coordinated interaction between autonomic and neuroendocrine responses (Morena et al. 2016). Stress stimulates the hypothalamic-pituitary-adrenal axis (HPA) to produce corticotrophin-releasing hormone (CRH) from paraventricular nucleus (PVN) of hypothalamus which fosters the anterior pituitary producing adrenocorticotropic hormone (ACTH) inducting glucocorticoid secretion (cortisol) to minimize immune responses (Hori and Kim 2019). Chronic stress is considered to jeopardize the progression of major depressive disorder (MDD), whereas exposure to acute yet extreme stressful events, often in individuals experiencing chronic stress, can precipitate the development of PTSD (Almeida et al. 2021).

A variety of studies have found hypocortisolism in people who have gone through a stressful event and then developed PTSD (Rohleder et al. 2004; Groer et al. 2015). Furthermore, expanding research has revealed that PTSD patients with hypocortisolism are more susceptible to developing proinflammatory cytokines (Daskalakis et al. 2016). Paraventriculare nucleus (PVN) neuronal activity in response to peripheral immunological challenge is mediated by cytokines-induced endothelial production of prostaglandins (Quan et al. 2003). This immune-induced HPA axis activation is primarily facilitated by prostaglandin E2 (PGE2) produced in the brain (Furuyashiki, Tomoyuki; Narumiya 2011). Notably, peripheral interleukin-1β (IL-1β) actuates the HPA axis, and increases ACTH release, likely through the initiation and release of CRH into the hypophyseal portal blood. PGE2 from endothelial cells of the brain microvasculature is also stimulated by peripheral IL-1β released from macrophages, which then acts PVN of the HPA axis (Parsadaniantz et al. 2000). Consequently, an increase in the release of CRH under chronic stress conditions and the further lack of a dearth of cortisol control over immune cells causes the, endothelial cells of PVN in the brain to mediate the immunological response and, exacerbating the PTSD condition.

The single prolonged stress (SPS) paradigm of PTSD in rats revealed that cyclooxygenase-2 (COX-2) can provoke inflammation and apoptosis in the hippocampus and contribute to the development of PTSD. Celecoxib, the selective COX-2 inhibitor decreased the levels of tumor necrosis factor- α (TNF-α), interleukin-6 (IL‐6), prostaglandin E2 (PGE2), and nitric oxide (NO) and hampered the neuronal apoptosis. Thus inhibition of COX-2 could decrease the occurrence of oxidative stress and apoptosis and can play a key role in clinical research and PTSD therapy in the future (Wang et al. 2018a). In LPS-challenged rats, doxycycline and meloxicam provided neuroprotection by lowering pro-inflammatory cytokine levels (TNF-α, IL-6, and IL-17) and COX-2 synthesis in the brain (Er et al. 2020). Moreover, studies using knockout mice and selective inhibitors have demonstrated that in a social defeat stress (SDS) model, toll receptors (TLR2/4), monoacylglycerol lipase (MAGL), and COX could induce PGE2 synthesis by TLR/MAGL/COX pathway causing social avoidance behavior (Nie et al. 2019).

As the global burden of Post-Traumatic Stress Disorder (PTSD) continues to rise and the disorder exhibits significant heterogeneity, it becomes crucial to comprehend the stress-related pathophysiology underlying PTSD. Though the existing research on the effectiveness of glucocorticoid-based treatment in preventing PTSD is encouraging, the mechanisms mediating this impact and the population that might benefit from this remains elusive due to methodological discrepancies and gaps in translational studies (Florido et al. 2023). Intervening in the established pathological pathways through repurposing drugs could offer a novel therapeutic approach to this complex condition (Table 1).

**Table 1:** Therapeutic strategies in PTSD research:

S.No	Neural systems and molecular markers	Observed dysregulation in markers/ receptors	DSM-V symptoms	Drugs	References
1	Serotonergic system	Low 5HT, Overactivity of 5HT_1A_, 5HT_1B_, 5HT_2A_, 5HT_2C_	Depressive like behavior, impulsivity, hypervigilance	Sertraline Paroxetine Fluoxetine	(Corchs et al. 2009; Luo et al. 2011; Baptista-de-Souza et al. 2020; de Moraes Costa et al. 2020)
2	Glutamatergic system	Increased glutamate	Hyperarousal, Intrusive memories	Ketamine NMDA receptor antagonist	(Rosso et al. 2017; Watson 2019)
3	GABAergic	Low GABA levels down-regulation of GABA_A_R	Anxiety, Intrusive memories	Gabapentin	(Nasca et al. 2013; Astill Wright et al. 2019)
4	Adrenergic	Increased NA	Hyperarousal	Prazosin, Clonidine Propranolol	(Olson et al. 2011; Aykac et al. 2020)
5	Cholinergic	Reduced Ach, Downregulated α7nAchR, increase AchE	Intrusive memories, negative mood	Donepezil, Cotinine	(Tyagi et al. 2010; Mendoza et al. 2018; Prajapati and Krishnamurthy 2021)
6	Dopaminergic	Low dopamine levels, decreased dopamine transporter (DAT), Decreased D_2_R expression D_3_R overexpression	Hyperarousal, negative mood	Asenapine, L-DOPA	(Cisler et al. 2020; Grinchii and Dremencov 2020)
7	Inflammasome	NLRP3	-	β-hydroxybutyrate (BHB)	(Yamanashi et al. 2020)
8	Sirtuins	Increases SIRT1, decreased SIRT6	-	EX527 (SIRT1 inhibitor)	(Li et al. 2019c)
9	Arachidonic acid metabolites	COX-2 PGE2	-	Meloxicam Ibuprofen Piracetam Celecoxib	(Mellon et al. 2018; Uniyal et al. 2019; Er et al. 2020)

## Channelization of Neural Systems Towards PTSD Symptomology

### Serotonergic System, Inflammation, and PTSD

The physiological involvement of the neurotransmitter serotonin (5-hydroxytryptamine; 5-HT) is essential for brain development, mood regulation, and stress reactivity (Brummelte et al. 2017), whereas, a deficiency plays a pivotal role in the pathophysiology of depression (Jacobsen et al. 2012; Yohn et al. 2017), attention deficit hyperactivity disorder (ADHD) (Whitney et al. 2016) and Alzheimer’s disease (Chakraborty et al. 2019). Majority of the serotonin receptors are metabotropic, and produce their physiological responses through the second messenger systems and are G-protein-coupled receptors (GPCRs), whereas 5-HT3 is an ionotropic receptor and acts through ligand-gated ion channel (LGIC) (Sarkar et al. 2021).

Disruption of 5HT transmission has been proposed to be involved in the etiopathogenesis of PTSD. Low hippocampal 5HT levels have been linked with the development of depressive-like behavior in the SPS-induced stress model of PTSD in rats (Sherin and Nemeroff 2011; Lee et al. 2020). The concentration of 5HT in the dorsal median raphe is also reduced, which may alter the dynamics between the amygdala and the hippocampus (Sukhmanjeet Kaur Mann; Raman Marwaha. 2021). These results are in line with the PTSD condition, where low 5HT levels result in depressive behavior, impulsivity, and hypervigilance (Nisar et al. 2020). Furthermore, the time-dependent sensitization (TDS) stress in rats has shown that the considerably reduced plasma corticosterone levels lead to quantitative (receptor density) and qualitative (receptor affinity) alterations in the hippocampal 5HT_1A_ and prefrontal cortex 5HT_2A_ receptors (Harvey et al. 2003). Moreover, the density of 5HT_1A_ receptors has also been shown to be upregulated in a rat model of PTSD suggesting the involvement of 5HT_1A_ receptors in modulating the anxiety phenotype of PTSD (Luo et al. 2011), and these outcomes were consistent with the neuroimaging findings in PTSD patients (Sullivan et al. 2013). Overactivity of 5HT_1B_ auto-receptors in dorsal raphe nucleus (DRN) neurons could be a key mediator of pathological reactions to stressful situations (Clark et al. 2002). In line with this, a genetic mouse model lacking 5-HT_1B_ autoreceptors demonstrated lower anxiety-like behaviour in the open field and antidepressant-like effects in the forced swim and sucrose preference tests. These findings imply that strategies aiming at inhibiting 5-HT_1B_ autoreceptors may be effective in the treatment of anxiety and depression which are the symptom clusters of PTSD (Nautiyal et al. 2016). The density of 5HT_1B_ receptors has also been related to certain PTSD characteristics, implying that certain aspects of the clinical phenomenology of PTSD may be caused by these receptor changes (Bailey et al. 2013; Pietrzak et al. 2013b). A case-control genotyping study in adults who had experienced significant trauma found a link between genetic disparity in the 5HT_2A_ promoter region and PTSD (Mellman et al. 2009). Further, chronic stress triggers the upregulation of 5HT_2C_ receptors which have been linked to altered neuroplasticity and neuroinflammation due to the elevation of IL-6, IL-1β and calcinurin in PTSD (Règue et al. 2019). The reduction of serotonin transporter (SERT) gene expression is linked with contextual fear memory extinction in the SPS PTSD rat model, indicating that SERT attenuation is associated with stabilization of 5HT levels and inhibits hippocampus autophagy (Wu et al. 2016).

**Fig. 1:**
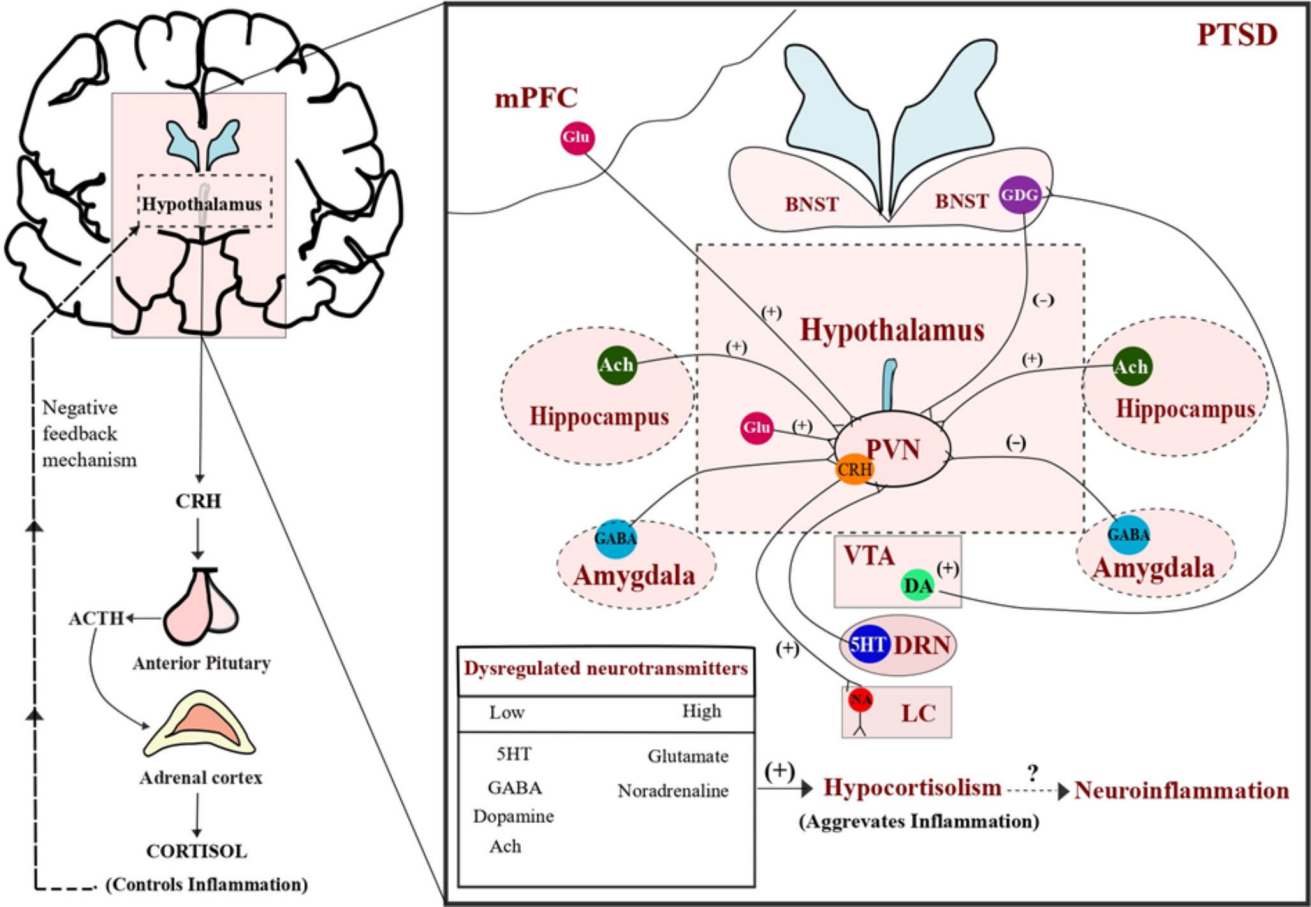
**Neural networks modulating HPA axis**: The stress response begins with the production of corticotropin-releasing hormone (CRH) from the HPA axis, which causes the anterior pituitary to produce adrenocorticotropic hormone (ACTH). Cortisol is then released by the action of ACTH on the adrenal cortex. It has negative feedback on the hypothalamus and anti-inflammatory effects. However, dysregulated neurotransmitters, along with hypocortisolism, may favour the development of neuroinflammation in PTSD (created by using Inkscape 1.2 version https://inkscape.org/)

The HPA axis stress response is modulated by 5HT inputs from the DRN (Tafet and Nemeroff 2016) (Fig. 1), and direct synaptic interaction of serotonergic axons with CRH neurons and mediate the release of ACTH and cortisol through activation of 5HT_1A_, 5HT_1B_, 5HT_2A,_ 5HT_2B,_ and 5HT_2C_ receptors in the hypothalamic PVN (Stephens and Wand 2012; Tung et al. 2012). The released cortisol has a detrimental impact on the DRN and 5HT-sensitive hippocampal neurons, which in turn results in low transcription of the gene encoding for 5HT_1A_ receptor. 5HT_1A_ auto receptor activation on the other hand results in DRN neuron hyperpolarization. This hyperpolarization decreases 5HT synthesis and altering the fear circuits and anxiety (Lanzenberger et al. 2010; Polter and Li 2010). Furthermore, corticosterone treatment in adrenalectomized rats has little effect on 5HT_1B_ mRNA levels in the dorsal raphe or hippocampus as compared to 5HT_1A_ mRNA (Neumaier et al. 2000). These results are significant since they hint at corticosterone’s selective modulation of receptors across brain areas. Evidence shows that genetic polymorphism in the central 5HT_2A/2C_ receptors results in abnormal cortisol secretion after impaired motor control and attention (Brummett et al. 2012; Murnane 2019). Alongside receptor overexpression or polymorphism, low platelet serotonin levels and blunted HPA axis activity is also attributed to suicidality behavior in PTSD patients (Grah et al. 2010). These pieces of evidence reveal that abnormal HPA axis activation together with dysregulated serotonergic system contributes to negative symptoms of PTSD.

**Fig. 2:**
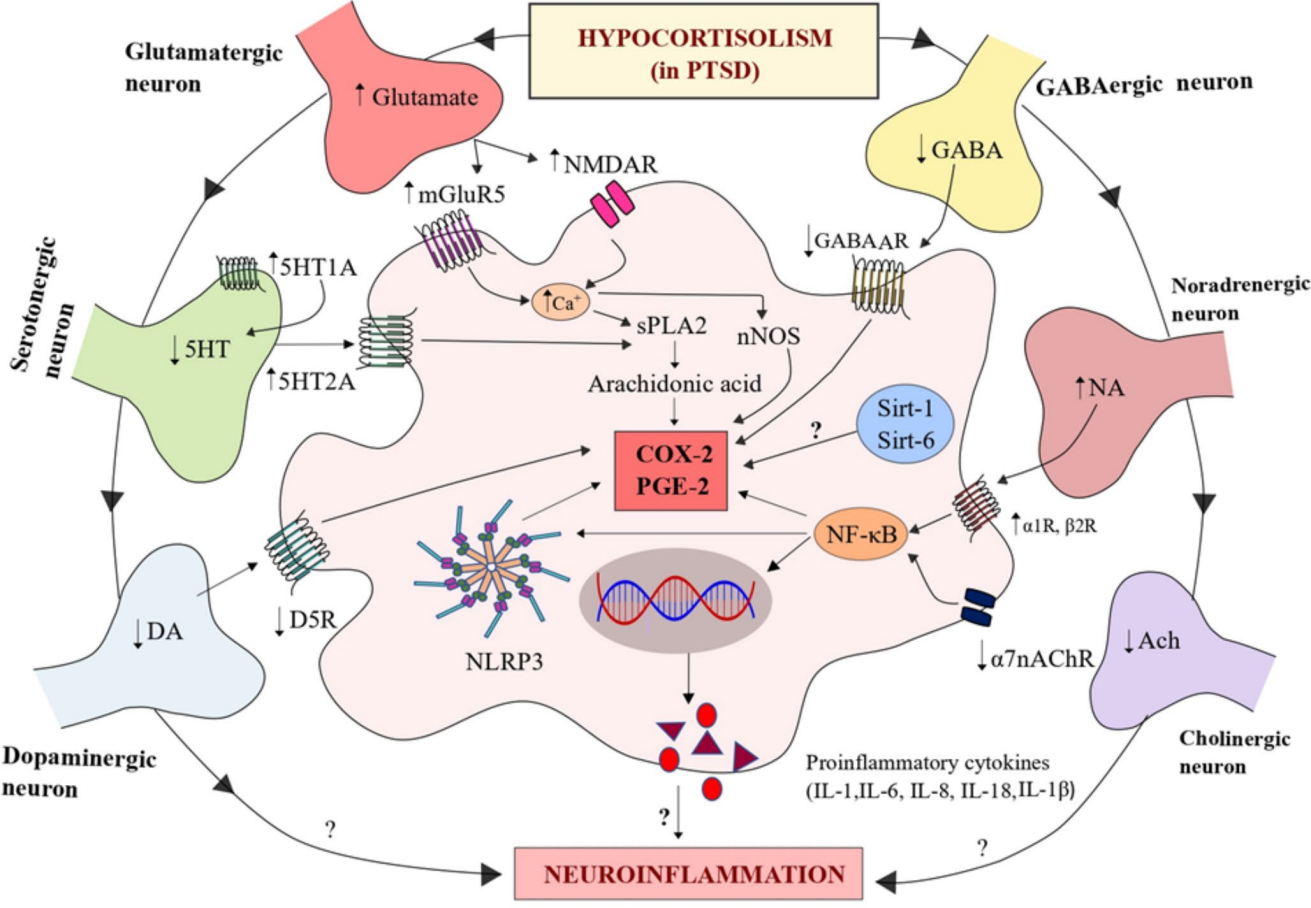
**Molecular mechanisms favoring neuroinflammation in PTSD**: In the PTSD condition, dysregulated neurotransmitters interact differently at the receptor level, promoting the synthesis of cyclooxygenase-2 (COX-2), prostaglandin E2 (PGE2) and other proinflammatory cytokines. Furthermore, NOD-like receptor family pyrin domain-containing protein 3 (NLRP3) and Sirtuins (SIRT-1 and SIRT-6) may have a role in aggravating the development of neuroinflammation in the PTSD condition (created by using Inkscape 1.2 version https://inkscape.org/)

**Fig. 3:**
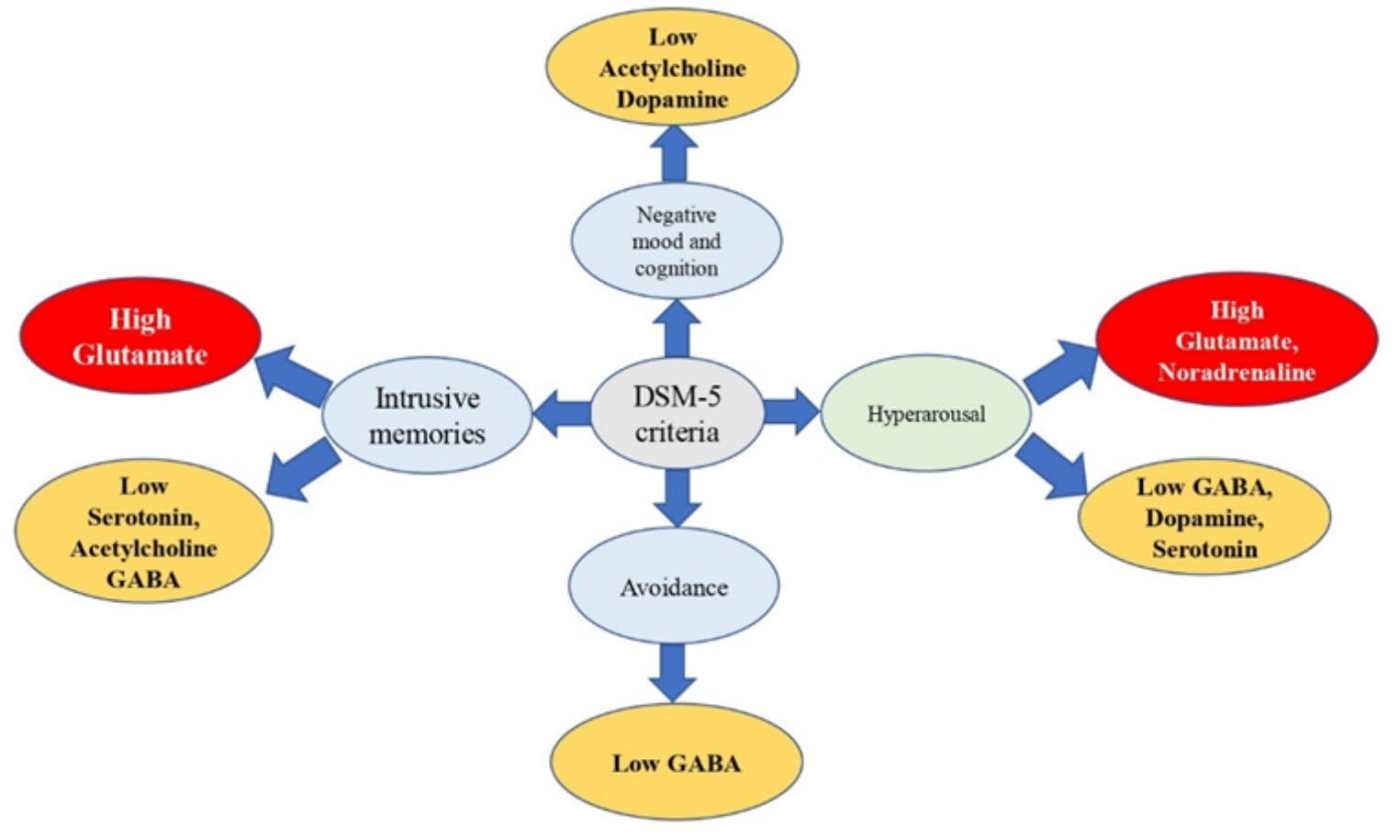
**DSM-V clusters and their relationship with neurotransmitters**: According to the Diagnostic and Statistical Manual of Mental Disorders (DSM-V), the four symptom clusters of PTSD include intrusive memories, avoidance of traumatic event reminders, negative mood and cognition, and hyperarousal. An imbalance between different neurotransmitters, such as excessive amounts of glutamate and noradrenaline and low levels of acetylcholine, γ-aminobutyric acid (GABA), dopamine, and serotonin encourage the development of these four PTSD symptom clusters (created by using Microsoft PowerPoint 2019)

Serotonin is involved in inflammatory and immunomodulatory disorders (Shajib and Khan 2015; Herr et al. 2017). Various *in-vivo* disease models and clinical investigations indicate that serotonergic transmission may influence the peripheral immune system. This raises serious concerns regarding the ability of various immune cells to produce, store, react to, and/or transport serotonin (Wu et al. 2019). The modulation of arachidonic acid (AA) turnover in the brain is instigated by 5HT via 5HT_1A_ receptors (Strosznajder et al. 1994; Gopaldas et al. 2019) (Fig. 2). Various lines of evidence pointed out that 5HT_2A_ receptor activation through Gα_12/13_ has been shown to control COX-2 activity as well as upsurge activation of serum-induced phospholipase A2 (PLA2) (Kurrasch-Orbaugh et al. 2003). Furthermore, selective 5HT_2A/2 C_ receptor agonist 1-[2,5-dimethoxy-4-iodophenyl]-2-aminopropane (DOI), has been shown to induce COX-2 activation in the rat parietal cortex (Mackowiak et al. 2002) (Fig. 2). Although serotonin deficiency is one of the hallmarks in the pathophysiology of PTSD, explicit research is anticipated to confirm the contribution of the serotonergic system in the significant inflammatory imbalance scenario of PTSD. Taken together, altered 5HT transmission may cause PTSD symptoms such as hypervigilance, heightened startle, impulsivity, and intrusive memories (Fig. 3), however, the precise roles and mechanisms remain elusive.

### Glutamatergic System, Inflammation, and PTSD

In the brain, glutamate is one of the major excitatory neurotransmitter and is at the crossroads of several metabolic pathways acts through both ionotropic (NMDA, AMPA, and kainate receptors), and/or metabotropic (mGluR_1 − 8_) glutamate receptors (Zhou and Danbolt 2014). Although most approaches indirectly evaluated glutamate neurosignaling, increasing evidence proposes glutamatergic dysfunction in several mental illnesses such as schizophrenia, Parkinson’s disease (PD), bipolar disorder (BD), major depressive disorder (MDD), obsessive-compulsive disorder (OCD), and PTSD (Li et al. 2019a; Nasir et al. 2020; Wang et al. 2020).

Glutamatergic signaling has a decisive role in the advancement of PTSD. Glutamate stimulates the release of CRH through moderation of neuronal inputs from the medial prefrontal cortex (mPFC) in the PVN of the hypothalamus, which modulates the HPA axis (Herman et al. 2002) (Fig. 1). Microinjections of glutamate into the rat PVN caused CRH and ACTH release, increased corticosterone levels, therefore favored arousal response by increasing the number of c-Fos positive CRH neurons (Kita et al. 2006). During the initial stage after trauma, elevated cortisol triggers the activation of NMDA receptors through NMDA-extracellular signal-regulated kinase (ERK) and mitogen and stress-activated kinase (MSK) (NMDA-ERK-MSK), and the glucocorticoid receptor (GR) pathways resulting in the formation of recurring memories, which is one of the phenotypes of PTSD (Fig. 3) (Reul and Nutt 2008). Neuroimaging studies and mGluR5 blockade studies have indicated that patients with PTSD overexpress mGluR5 and which is linked with suicidal ideation and avoidance symptoms (Holmes et al. 2017; Davis et al. 2019). In the SPS rat paradigm of PTSD, it was concluded that increased anxiety and impaired fear memory extinction were due to the overactivity of glutamatergic neurons with the subsequent difference between excitatory and inhibitory neurotransmission in the amygdala and this imbalance was correlated to the development of PTSD (Fang et al. 2018). These conclusions are in line with serum analysis and neuroimaging studies in PTSD patients, emphasizing the need for novel PTSD therapies that target the glutamatergic system. (Nishi et al. 2015; Harnett et al. 2017; Rosso et al. 2017).

A randomized controlled trial involving individuals who had surgery for minor head injuries revealed that selective COX-2 inhibition had a brain-protective effect via reducing glutamate levels (Bisri et al. 2017). In vitro studies also confirmed that glutamate NMDA receptor coupling results in Ca^+^ entry and propels neuronal nitric oxide synthase (nNOS) activation. The binding of nNOS to COX-2 results in NMDA-mediated excitotoxicity and also the formation of prostaglandins (Fig. 2). These outcomes suggest that the drugs inhibiting nNOS-COX-2 binding might lower prostaglandin levels in the brain, hence reducing excitotoxicity and neural dysfunctions. Secretory PLA2 (sPLA2) is stored in the synaptic vesicles and discharged in response to neural stimulation. *In-vivo* and *in-vitro* studies highlighted that both glutamate and sPLA2 increased the expression of neuronal COX-2, and these results indicate that COX-2 expression mediated by sPLA2 and glutamate results in the activation of the arachidonic acid paving the way for excitotoxicity-mediated neuroinflammation (Kolko et al. 1996, 2002).

### GABAergic Neurotransmission and PTSD

GABA (gamma-aminobutyric acid) is a major inhibitory neurotransmitter in the CNS and is involved in regulating various physiological and pathophysiological pathways in the brain and peripheral tissues. GABA is largely produced from glutamine and glutamate by the action of glutaminase and glutamate decarboxylase (GAD) respectively (Watanabe et al. 2002). GABAergic neurons can be found in the hippocampus, thalamus, basal ganglia, hypothalamus, and brainstem. For optimal cell membrane integrity and neurologic function, a balance of inhibitory and excitatory neurotransmission via GABA and glutamate is required (Allen J, Mary; Sabir, Sarah; Sharma 2021).

GABAergic neurotransmission disruption may trigger PTSD pathogenesis (Arditte Hall et al. 2021) where the involvement of GABA_A_ receptors is proven to inhibit hyperarousal state and anxiety. In juvenile rats, inescapable foot shock caused persistent anxiety, spatial memory loss, and decreased GABA_A_R subunit expression. Low brain levels of GABA are consistent with the overall findings in anxiety disorders and the hyperarousal hypothesis of both primary insomnia and PTSD (Meyerhoff et al. 2014). Low plasma concentration of GABA was seen in PTSD patients who also had symptoms of anxiety, avoidance, and hyperarousal suggesting that estimation of GABA levels might be used as a biomarker to assess PTSD severity (Fig. 3) (Trousselard et al. 2016). These results correspond to prior human neuroimaging research that indicates aberrant glutamate and GABA amounts in the brains of PTSD patients (Markus et al. 2016; Sheth et al. 2019).

While CRH neurons get inputs from a variety of brain regions, they are ultimately regulated by GABAergic inhibition from the amygdala (Myers et al. 2014) (Fig. 1) mediated by GABA_A_R on CRH neurons (Mody and Maguire 2012; Errington 2014). Electrophysiological recordings and *in-vitro *studies of CRH neurons in rodent hypothalamic brain slices have demonstrated that corticosteroids augment extrasynaptic GABA_A_R-mediated tonic currents (Herman et al. 2004; Colmers and Bains 2018). Moreover, selective deletion of the GABA_A_ α1 subunit gene in the CRH neurons of mice produces a phenotype with increased anxiety as well as impaired fear memory extinction, both of which are hallmarks of PTSD (Gafford et al. 2012). Apart from differences in GABA_A_R expression, recent research indicates that the GABAergic neurotransmission in response to stress is more complicated, involving changes in chloride homeostasis as well as synaptic plasticity. Hence in the chronic stress disorders like PTSD, a condition of hypocortisolism, elevated CRH levels, downregulation of GABA_A_R with a substantial reduction in GABA levels favors hyperarousal (Fig. 3) and anxiety phenotypes of PTSD.

Activation of cyclooxygenases (COXs) may produce an increase in free radical generation, resulting in oxidative stress and apoptosis of GABAergic neurons and hence a rise in glutamate activity. Celecoxib, a selective COX-2 inhibitor has been shown to increase the expression of GABA_A_ receptors and thereby increase the fast inhibitory neurotransmission in the hippocampus (Haiju et al. 2009). Whole-cell patch-clamp observations from parvocellular neuroendocrine cells (PNCs) of the hypothalamus in rats revealed that PGE2 inhibits the release of GABA onto para neuroendocrine cells (PNCs) in the PVN by presynaptic EP3 receptors, suggesting a possible mechanism by which local PGE2 activity in the PVN modulates the HPA axis during inflammation (Khazaeipool et al. 2018). Ferri and Ferguson in 2005 have shown that PNC cells in the PVN were depolarized by both IL-1β in a COX-2-dependent PGE2 stimulation, and the effect was dependent on the reduced GABAergic input caused by direct hyperpolarization of these neurons in the halo zone surrounding and projecting to the PVN (Ferri and Ferguson 2005) (Fig. 2). These evidence support the involvement of inhibitory amino acids involvement in PTSD and further detailed studies will add on to the existing understanding of this concept.

### Dopaminergic System, Stress, Inflammation, and PTSD

Dopamine (DA) is a neurotransmitter that regulates motor control, motivation, reward, and cognitive function in CNS (Klein et al. 2019). In the mesocorticolimbic dopaminergic pathway, the synthesis of DA takes place in the midbrain ventral tegmental area (VTA) and is released into the nucleus accumbens (NAcc) and the medial prefrontal cortex (mPFC) (Juárez Olguín et al. 2016). All the dopaminergic receptors are metabotropic and D_1_, D_2_, D_3_, D_4_, and D_5_ receptors are linked to Gαs and Gαi G-proteins (Zhou et al. 2021).

Various studies have demonstrated that aberrant dopaminergic transmission from VTA to mPFC and hippocampus significantly contributes to PTSD symptoms including maladaptive memory consolidation (Torrisi et al. 2019; Zhou et al. 2021). Furthermore, the genetic alterations in the DA reuptake protein, dopamine transporter (DAT) could pave the way for PTSD condition (Drury et al. 2013; Zuschlag et al. 2021). Homozygous DAT gene ablation in rats showed neurodegeneration and glial cell activation providing new acumen into the association of DAT in the neuroinflammatory process (Illiano et al. 2021). Recently Yuan et al., (2021) have shown that Taq1A polymorphism of the DA D_2_ receptor (D_2_R), where the T allele carriers of D_2_R Taq1A express lower D_2_R density, higher levels of neuroinflammation and hippocampal atrophy and this causes reduced hippocampal subfield volume of CA3 region and severe PTSD symptoms (Yuan et al. 2021). In combat veterans with PTSD, the D_2_R gene is specifically linked to comorbid severe anxiety, depression, and social dysfunction (Lawford et al. 2006). Using a genome-wide DNA methylation pattern in a three-year follow-up study of veterans, it was shown that the Dopamine-PKA-CREB signaling pathway is often dysregulated and highly related to the hyperarousal phenotype in PTSD (Fig. 3) (Yang et al. 2020). Furthermore, deletion or antagonism of D_3_R resulted in anxiolytic effects in the rodent SPS model of PTSD, implying that D_3_ receptor antagonism was effective in reducing PTSD symptoms and also could decrease the risk of drug abuse and addiction (Rice et al. 2018; Song et al. 2018).

Although there are no direct dopaminergic projections from the VTA to the PVN, DA afferents from the VTA to the dorsolateral bed nucleus of stria terminalis (dlBNST) are crucial for the control of the HPA axis inhibiting CRH release (Di et al. 2020) (Fig. 1). Usually, under stress conditions, glucocorticoids stimulate DA release and generate euphoric emotions and movements in the mesolimbic dopaminergic regions (Butts and Phillips 2013; Howes et al. 2017). Various animal and clinical evidence emphasized that chronic stress impairs working memory via a significant reduction in DA concentration in the striatum and PFC (Mizoguchi et al. 2000; Bloomfield et al. 2019). Similarly, in the electric foot shock stress-restress exposure model of PTSD, it was shown that reduced hypothalamus and pituitary corticosterone levels, increased CRH, and glucocorticoid receptor gene expression (GR) were responsible for the reduced sniffing, rearing, and grooming activities in rats (Asalgoo et al. 2017). Furthermore, in SPS-induced stress in rats, low corticosterone is attributed to a decrease in DA levels along with elevated oxidative stress and neuroinflammation in cortical and hippocampal brain areas, which contributes to the clinical progression of PTSD (Uniyal et al. 2019). These findings imply that hypocortisolism and hypodopaminergia could potentially contribute to the negative mood and hyperarousal symptoms connected with PTSD (Fig. 3).

DA has been shown to modulate systemic inflammation through D_1_R signaling and acts as an endogenous inhibitor of the NLRP3 inflammasome activation pathway (Northrop and Yamamoto 2013). This suggests that DA may function as a coping mechanism against the development of inflammatory disorders and that D_1_R is a possible therapeutic target for NLRP3-driven inflammatory diseases (Yan et al. 2015; Wang et al. 2018b). Inflammatory cytokines have been shown to directly target DA and reward circuitry, contributing to depressive symptoms such as emotional exhaustion and motor retardation. Inflammatory cytokines appear to affect various elements of DA neurotransmission, resulting in reduced synthesis and/or defective packing or release, all of which may combine to varying degrees to lower DA function (Felger 2016). According to the current evidence, high DA levels activate low-affinity DA receptors (including D_1_R, D_2_R, and D_4_R), which have an anti-inflammatory impact on the cells of the immune system, but low DA levels precisely activate high-affinity DA receptors (including D_3_R and D_5_R), which causes neuroinflammation (Fig. 2). Under chronic stress conditions, as depicted in the chronic unpredictable stress paradigm in rats, COX activity caused an increase in striatal DAergic damage (Northrop and Yamamoto 2013). COX-2 is generally expressed at low levels in nigral DAergic neurons but it is up-regulated under both clinical and experimental conditions of Parkinson’s disease (Fathi Moghaddam et al. 2008). In animal models of Parkinson’s disease, COX-2 activation amplifies the cytotoxic effect by the activation of microglia and produces pro-inflammatory prostaglandins, iNOS, ROS and neurodegeneration (Chauhan et al. 2018; Ardah et al. 2020). Moreoveor, Carrasco et al., (2005) have shown that challenge of mesencephalic neuronal cultures by 6-OHDA and MPP + revealed, that exposure to 6-OHDA rather than MPP + in 24 h increased COX-2 dependent prostaglandin levels and Ibuprofen (COX inhibitor) suppressed PG rise and was inversely associated with dopaminergic cell death (Carrasco et al. 2005). COX-2 inhibition may protect from neuronal injury through microglia-independent mechanisms such as COX-2-mediated DA oxidation to quinone species, which produces oxidative stress and neuroinflammation (Chae et al. 2008; Vidal and Pacheco 2020). These data reinforce the use of anti-inflammatory drugs to treat neurodegenerative maladies involving DA and stress.

### Adrenergic Neural Network and PTSD

The sympathetic nervous system controls a variety of biological activities including the regulation of the immune system (Sharma and Farrar 2020). Most of the noradrenergic neurons in CNS are localized in the brainstem nucleus, locus coeruleus (LC) (Berridge and Waterhouse 2003). LC neurons are involved in neuromodulation through neuronal inputs to the prefrontal cortex (PFC), basolateral amygdala (BLA), and motor cortex (Chandler et al. 2019). Noradrenaline (NA) release during acute stress generates a state of alertness and facilitates sensory processing to boost memory consolidation throughout stressful situations (Daviu et al. 2019).

Trauma and/or long-term stressors might produce dysregulation in noradrenergic neural networks, which has been implicated in the pathophysiology of PTSD as the source of hyperarousal clusters (Nwokafor et al. 2021). Neuroimaging findings in PTSD patients revealed that behavioral and autonomic hyper-responsiveness is induced by a strong phasic noradrenergic stimulus originating in the LC (Naegeli et al. 2018). Furthermore, cerebrospinal fluid (CSF) NA levels were shown to be high and positively linked with the severity of PTSD symptoms (Baker et al. 2001). There is an increased presynaptic outflow along with increased postsynaptic responsiveness to NA in CSF of PTSD patients (Geracioti et al. 2001). *In-vivo* studies and PET imaging in PTSD patients have indicated that considerable changes in norepinephrine transporter (NET) levels in the LC are related to an increase in the intensity of arousal symptoms (Pietrzak et al. 2013a; Sabban et al. 2018). In rodents, exaggerated acoustic startle response and reduced locomotor activity in a novel atmosphere have been utilized as indices of hyperarousal after a traumatic experience. Inescapable foot shock (IFS) enhanced the stress-induced extracellular concentration of NA in the amygdala and diminished locomotion as an indicator of hyperarousal in the PTSD condition (Jacek Dębiec, David E. A. Bush 2014; Ronzoni et al. 2016).

The release of CRH from CRH-containing terminals in the LC stimulates NA release (Jedema and Grace 2004) (Fig. 1). While increased noradrenergic activation of hypothalamic PVN may explain why CRH levels in PTSD patients are high. These data suggest that both acute and persistent upsurge in CRH outflow to the LC can boost noradrenergic outflow (O’Donnell et al. 2004). Further activation of CRH receptors may be altered by repeated stress-induced NA release (Rajbhandari and Bakshi 2020). Convergent models of PTSD show that cortisol and NA release leads to more intrusive memories in PTSD and the combination of NA and cortisol substantially predicts intrusive memories in PTSD patients. These findings imply a strong correlation between stress hormones and memory consolidation in PTSD requires a state of heightened arousal (Fig. 3) (Nicholson et al. 2014; Mather et al. 2016). Various clinical studies pointed out that patients with PTSD exhibited considerably increased NA secretion and decreased cortisol levels (Pervanidou 2012; Wingenfeld et al. 2015). This amplified NA release can provoke the synthesis of proinflammatory cytokines such as IL-1 and IL-6 via nuclear factor-κB (NF-κB)-dependent processes. Further excessive noradrenergic stimulation potentiates fear conditioning by inducing calcium influx in astrocytes via adrenergic receptors (Gazarini et al. 2013). Cortisol hinders sympathetic nervous system (SNS) hyperactivity via suppressing NF-κB signaling, which can reduce the production and release of proinflammatory cytokines (Tan et al. 2007). However, a persistent state of hypocortisolism in people with PTSD may lead to SNS hyperactivity, which accelerates inflammation.

In rats, ICV administration of arachidonic acid raised adrenaline and noradrenaline plasma levels after 20–30 min signifying that instigation of the brain phospholipase A_2_-arachidonic acid cascade promotes central sympatho-adrenomedullary outflow (Yokotani et al. 2000). Centrally administered CRH also enhances the expression of COX-1 and COX-2 in spinally projecting PVN neurons and COX-2 in LC neurons, indicating that COX isozymes are implicated in CRH-induced sympathetic modulation in rats (Yamaguchi and Okada 2009). Emerging evidence also suggests that PGE2 modulates sympathoexcitatory actions that are primarily arbitrated by the EP3 receptor (Zhang et al. 2011; Shimizu et al. 2014). These findings imply that the central excitatory effects of COX-2 and PGE2 on PVN neurons, in tandem with heightened sympathetic activity, hypocortisolism, and inflammation, may pave the way for neuroinflammation in PTSD.

### Cholinergic System, Cognitive Inflexibility, and PTSD

Cholinergic signaling is critical for cognitive function, and its dysfunction is a hallmark of many neurodegenerative disorders, including Alzheimer’s disease (Hoskin et al. 2019; Winek et al. 2021). Furthermore, in various rodent stress models, cholinergic neurotransmission has been shown to play a crucial role in learning and memory extinction. (Srikumar et al. 2006; Yanpallewar et al. 2022). The hippocampus is enriched in cholinergic innervation and plays a pivtol role in cognitive function and stress-related behaviour (Pavlovsky et al. 2012). Acetylcholine (ACh) elicits its action through nicotinic and muscarinic receptors (Tiwari et al. 2013). Hence nicotine-based compounds have been proposed as potential therapeutical tools for the treatment of PTSD (Barreto et al. 2015). Cotinine, an active metabolite of nicotine has drawn significant attention in the recent past as a potential positive modulator of α7 nicotinic acetylcholine receptor (α7nAChR), and in a mice PTSD model, it actively enhances the fear extinction and reduces anxiety and depressive behaviour in an α7nAChR-dependent manner (Barreto et al. 2015; Mendoza et al. 2018; Aliev et al. 2020). Cotinine modulates synaptic plasticity and PTSD symptoms by stimulating downstream signaling of α7nAChR receptor; the protein kinase B (Akt)/glycogen synthase kinase 3β (GSK3β) pathway and ERKs (extracellular signal-regulated kinases) (Barreto et al. 2015; Mendoza et al. 2018). Furthermore, activation of α7nAChR modulates inflammatory pathways like, TLR4/NF-κB inflammasome and mTOR mediated autophagy and reduces pro-inflammatory cytokines (IL-6, IL-1β, TNF-α) (Bencherif et al. 2011; Ke et al. 2017). Consistent with these findings cotinine, a nootropic agent that modulates α7nAchR could be used as adjunctive therapy for PTSD (Table 1) and other neuropsychiatric conditions that cause neuroinflammation and dysfunction of learning and memory (Fig. 3) (Mendoza et al. 2018).

Reduced cognitive flexibility has recently been linked to predicting PTSD symptoms, where low flexibility has been suggested to be a risk factor for more severe PTSD symptoms (Ben-Zion et al. 2018). The cholinergic deficit has also been reported in the SRS-induced PTSD model in rodents, where donepezil ameliorated the SRS-induced cognitive inflexibility, downregulation of α7nAChR and expressed a reduced activity of choline acetyltransferase (ChAT) along with the increased activity of acetylcholine esterase (AChE) enzymes respectively (Prajapati and Krishnamurthy 2021). AChE activity in the basolateral amygdala in rats altered the strength or duration of cholinergic transmission during fear extinction (Kellis et al. 2020). In PTSD patients, single-photon emission computed tomography (SPECT) revealed that high concentrations of β2nAChRs in the thalamus promote re-experiencing symptoms through altering sensory input to the cortex and cortical neuroplasticity related to learning and stress response (Czermak et al. 2008). Various studies have also indicated that activation of α7nAChR lowers the levels of pro-inflammatory mediators and has a high potential to lower a variety of inflammatory-mediated ailments and neurological disorders, including PTSD (Bencherif et al. 2011; Sun et al. 2017; Ke et al. 2017). Inflammation, reduced baroreflex sensitivity (BRS), diminished parasympathetic nervous system (PNS), and excessive sympathetic nervous system (SNS) activity is proposed as contributory mechanisms for the severity of PTSD in a study including military veterans (Ulmer et al. 2018; Fonkoue et al. 2020).

Emerging data suggest that CRH modulates cognitive functions that rely on the cholinergic basal forebrain (Hupalo et al. 2019) through CRH1R present on cholinergic neurons and facilitates acetylcholine release (Day et al. 1998; Sauvage and Steckler 2001). Also, stress-induced responses activate the septohippocampal cholinergic pathway, which eventually activates the HPA axis (Paul et al. 2015) (Fig. 1). In bovine adrenal zona fasciculata/reticular (ZFR) cells, it was revealed that acetylcholine governs cortisol release at the cellular level via muscarinic M_3_ receptor (M_3_R) connected to phospholipase C (Walker et al. 1990). Stress in rodents enhanced the release of acetylcholine in the limbic areas (Imperato et al. 1989) and the HPA axis has been attributed to the susceptibility of basal forebrain cholinergic nerve cells (Aisa et al. 2009). Interestingly, Donepezil (AChE enzyme inhibitor) increased ACh availability, lowering negative symptoms in PTSD patients, and also inhibited LPS-induced neuroinflammation via α7nAChRs, which is followed by the PI3K-Akt mechanism, and this pathway might serve as a reference for the emergence of new therapies for reversing neuroinflammation or offer new indications for existing treatments (Table 1) (Tyagi et al. 2010; Navarro et al. 2021; Prajapati and Krishnamurthy 2021).

Cholinergic neurosignaling influences immune cell proliferation, cytokine production, T helper differentiation, and antigen presentation. These effects are facilitated through cholinergic muscarinic and nicotinic receptors and other cholinergic constituents found in immune cells, such as AChE and ChAT. Acetylcholine protects neurons from LPS-induced neuronal damage by suppressing the inflammatory response in rats (Li et al. 2019b). The anti-inflammatory mechanism favored by α7nAChR activation occurs through recruitment and stimulation of the Jak2/STAT3 pathway, which suppresses NF-κB nuclear translocation (Fig. 2) while activating the master regulator of oxidative stress Nrf2/HO-1 (Egea et al. 2015; Patel et al. 2017). Recent rodent experiments revealed that activation of α7nAChR reduced COX-2 expression, microsomal prostaglandin E synthase-1 (mPGES-1), and secretion of PGE2 (Piovesana et al. 2021; Peng-Fei et al. 2021). Nevertheless, in PTSD, hypoactivity of the cholinergic system with downregulated α7nAChR receptors could favor the negative mood and cognition aggravated with an inflammatory condition (Fig. 2).

## NLRP3 Inflammasomes and PTSD

Emerging evidence of research emphasized the menacing role of NOD-like receptor family pyrin domain-containing protein 3 (NLRP3) in the etiology of many neurodegenerative disorders (Holbrook et al. 2021), traumatic brain and spinal cord injury (Zhou et al. 2022) and neuroinflammation (Lin and Mei 2021). The NLRP3 inflammasome is a multimeric protein complex that initiates pyroptosis and causes proinflammatory cytokines to be released (Yang et al. 2019b). It is made up of a sensor (NLRP3), an adapter (ASC; also known as PYCARD) and an effector (caspase 1). NLRP3 contains amino-terminal pyrin domain (PYD), a core NACHT domain (Swanson et al. 2019), combined with the adapter molecule apoptosis-associated speck-like protein comprising CARD (ASC) to recruit the effector caspase-1 to allow the IL-1 family cytokines IL-1β and IL-18 to be proteolytically cleaved. TLR agonists induce NF-κB-mediated NLRP3 formation and pro-IL-1β expression (priming phase) along with ATP, K^+^ ionophores, heme, and pathogen-associated RNA promotes NLRP3 inflammatory assembly (activation) caspase-1-mediated IL-1β, IL-18 secretion, and pyroptosis (Yang et al. 2019b). In addition to these upstream activities, a variety of NLRP3-interacting proteins and posttranslational changes to NLRP3 often control inflammatory activation of NLRP3 (Duan et al. 2020).

There has been limited literature supporting the direct involvement of NLRP3 in PTSD. However, it has recently been shown in the SPS-induced PTSD rat model that inhibition of NLRP3 inflammasome activity by using an endogenous inhibitor, β-hydroxybutyrate (BHB) produces anxiolytic effects and reduced stress-induced TNF-α levels (Yamanashi et al. 2020). These findings imply that administering BHB might effectively tackle the inflammatory pathways linked with PTSD (Yamanashi et al. 2020). Similarly, deletion of the NLRP3 gene in mice demonstrated that the NLRP3 inflammasome was stimulated in the hippocampus 72 h following electric foot shocks in a contextual fear paradigm, which was accompanied by an increase in the toll-like receptor, retinoic acid-inducible gene (RIG-I) like receptor signaling, and a decrease in post synaptic density (PSD)-related proteins. Both genetic deletion and pharmacologic blockade of the NLRP3 inflammasome may improve extinction of contextual fear memory and reduce anxiety-like behavior, offering a novel therapy for trauma and stress-related disorders such as PTSD (Dong et al. 2020). *In-silico* and *in-vitro* studies employing phenylpropanoids performed in our lab, have yielded encouraging results against neuroinflammation. The compounds studied strongly suppressed the NLRP3 inflammasome pathway in glial cells, as shown by mRNA levels of key proteins and IL-1β production (Kinra et al. 2021).

*In-vitro* and *in-vivo* studies conducted by (Feng et al. 2019) revealed that chronic stress activates the GR-NF-κB-NLRP3 signaling in microglia, causing hippocampal neuroinflammation and depression-like behavior. Chronic stress also causes glucocorticoid resistance as observed in PTSD permits proinflammatory signaling pathways markedly by IL-1β which is a byproduct of NLRP3 activation, to bypass normal feedback control. The surge in IL-1β, in particular, may not be counteracted by a deficiency of cortisol, and this incidence might destabilize the CNS (Zefferino et al. 2021). NLRP3 is also engaged in serotonergic (Iwata et al. 2013), Glutamatergic (Yang et al. 2019a), GABAergic (Zhang et al. 2013; Xia et al. 2021), Dopaminergic (Yan et al. 2015), Adrenergic (Horstmann et al. 2016) and cholinergic (Ke et al. 2017; Wei et al. 2019) systems of CNS.

IL-1β can stimulate gene expression and production of COX-2 and PGE2 (Dinarello 2009). It has been proposed that COX-2 has a crucial role in PGE2-induced activation of the NLRP3 inflammasome, which is mediated via activation of NF-κB (Fig. 2) and caspase-1, as well as the release of mtDNA and mtROS. Furthermore, in response to the LPS-challenge, COX-2 inhibition in mice with celecoxib lowered IL-1β and caspase-1 in the spleen and liver. These findings provide novel insights on how COX-2 controls NLRP3 inflammasome activation and indicate that it might be a novel potential therapeutic target (Table 1) in NLRP3-related illnesses (Zhang et al. 2018; Hung et al. 2019).

## Sirtuins and PTSD

Sirtuins (SIRTs) are ubiquitous regulators of cell activities that are class III histone deacetylases and have been shown to have neuroprotective properties in a variety of neurodegenerative disorders (Liu et al. 2021; Ranadive et al. 2021). In PVN, SIRT1 stimulates the HPA axis and basal glucocorticoid (GC) levels by increasing CRH production via an increase in prohormone convertase 2 (PC2) biosynthesis, which is required for the maturation of CRH from pro-CRH (Toorie et al. 2016; Yamamoto and Takahashi 2018). SIRT1 induces deacetylation of helix loop helix transcription factor 2 (NHLH2) in the ventral CA1 region of the brain and enhances MAO-A transcription which then results in decomposition of serotonin (5HT) to 5 hydroxy indole acetic acid (5-HIAA) and influences PTSD-like moods and behaviors in SPS model of PTSD. While SIRT1 knockout mice and its inhibitor exhibited diminished anxiety and fear memory behaviors following the SPS procedure. These findings suggested that SIRT1 may be associated with the development of PTSD-like symptoms in reaction to extreme stress (Libert et al. 2011). Enhanced contextual memory is persistently noticed in PTSD (Al Abed et al. 2020). In the brain, ventral CA1 (vCA1) hippocampal projections deliver aversive stimuli-related inputs to the basal amygdala (BA), which serves to encode conditioned fear memory (Kim and Cho 2020). Furthermore, loss of SIRT6 in neuronal progenitors leads to tau-protein accumulation and loss of associative and non-associative memory, whereas overexpression of SIRT6 impairs long-term contextual fear memory by hindering IGF/Akt signalling pathway, that stimulates cAMP response element-binding protein (CREB). This pathway may be activated and contributed to the increase of contextual fear memory (Yin et al. 2016; Kaluski et al. 2017). Interestingly, genetic SIRT6 depletion in excitatory neurons, showed significant elevated contextual fear memory while spatial memory was not effected suggesting that enhancement in negative memory was due to reduced SIRT6 activity (Kim et al. 2018).

In the human umbilical vein endothelial cells (HUVECs), SIRT6 overexpression was associated with reduced NF-κB transcriptional activity, whereas knockdown of SIRT6 boosted NF-κB expression and resulted in COX-2, PGE2, and pro-inflammatory cytokines production (IL-6, IL-8) (Fig. 2). The overall outcomes of this study reveals that the loss of SIRT6 in endothelial cells is connected with an increase in the expression of genes implicated in inflammation (Lappas 2012). Although sirtuins are involved in alleviating neurodegenerative disorders (Yeong et al. 2020), SIRT 1 paradoxically increases the risk of PTSD while SIRT6 indirectly reduces PTSD symptoms. These findings further warrant more research to establish the detrimental or protective role of sirtuins in PTSD.

## Conclusion

The pharmacological treatment of PTSD has been restricted due to the narrow focus on the monoamine system and a lack of efficacy with current treatment approaches, indicating a gap in translating basic research to clinical research. Thus, future research on the pharmacological treatment of PTSD should not only concentrate on novel neurotransmitter pathways but also aim to enhance the understanding of the pathophysiology of the cognitive and emotional processes involved in PTSD. This will help to fully restore functions rather than merely compensating for posited deficits, leading to therapeutic innovations in the field of PTSD. This review summarises the psycho-neuro-immunological interplay in PTSD which could pave the way for neuroinflammation. Hence parallel to mechanistic research, efforts should be aimed at identifying novel pathways that would unravel the new treatment outcome is an emerging challenge that could lead to effective methods of preventing and treating PTSD.

## Data Availability

Not applicable.
